# Long-term outcomes of third-line therapy with tyrosine kinase inhibitors in chronic phase chronic myeloid leukemia: A real-life experience

**DOI:** 10.3389/fonc.2023.1138683

**Published:** 2023-03-16

**Authors:** Tamara Chitanava, Iuliia Matvienko, Vasily Shuvaev, Sergey Voloshin, Irina Martynkevich, Yulia Vlasova, Elizaveta Efremova, Ekaterina Mileeva, Anna Pirkhalo, Taiana Makarova, Roman Vlasik, Elena Karyagina, Natalia Il`ina, Nadezhda Medvedeva, Natalia Dorofeeva, Tatiana Shneider, Nadia Siordiya, Olga Kulemina, Evgenia Sbityakova, Natalia Lazorko, Julia Alexeeva, Dmitrii Motorin, Elena Morozova, Elza Lomaia

**Affiliations:** ^1^ Research Department of Immuno-Oncology, Almazov National Medical Research Centre, Saint Petersburg, Russia; ^2^ Clinical and Diagnostic Department of Hematology and Chemotherapy with a Day Patient Facility, Russian Research Institute of Hematology and Transfusiology, Saint Petersburg, Russia; ^3^ Clinical Department of Hematology, Chemotherapy and Bone Marrow Transplantation with Intensive Care Unit, Russian Research Institute of Hematology and Transfusiology, Saint Petersburg, Russia; ^4^ Research Center of Cellular and Molecular Pathology, Russian Research Institute of Hematology and Transfusiology, Saint Petersburg, Russia; ^5^ Bone Marrow Transplantation Department for Adults, Raisa Gorbacheva Memorial Research Institute of Children's Oncology, Hematology and Transplantation First I. Pavlov State Medical University of St. Petersburg, Saint Petersburg, Russia; ^6^ Department of Oncohematology, Chemotherapy and Bone Marrow Transplantation №11, City Clinical Hospital №15, Saint Petersburg, Russia; ^7^ Trans-Regional Hematology Department, City Clinical Hospital №15, Saint Petersburg, Russia; ^8^ Center for Outpatient Oncological Care, City Clinical Hospital №31, Saint Petersburg, Russia; ^9^ Department of Chemotherapy and Bone Marrow Transplantation №2, Almazov National Medical Research Centre, Saint Petersburg, Russia; ^10^ Department of Specialized Medical Care for Oncology Patients, Almazov National Medical Research Centre, Saint Petersburg, Russia; ^11^ Department of Oncology Hematology and Transplantation for Adolescent and Adults, Raisa Gorbacheva Memorial Research Institute of Children's Oncology, Hematology and Transplantation First I. Pavlov State Medical University of St. Petersburg, Saint Petersburg, Russia; ^12^ Department of Faculty Therapy with Clinic, Almazov National Medical Research Centre, Saint Petersburg, Russia

**Keywords:** chronic myeloid leukemia, chronic phase, efficacy of therapy, complete cytogenetic response, third-line therapy

## Abstract

**Introduction:**

Tyrosine kinase inhibitor (TKI) therapy has greatly improved the prognosis of patients with chronic myeloid leukemia (CML), improving the survival expectancy of patients with chronic phase (CP) CML to that of the general population. However, despite these advances, nearly 50% of patients with CP CML experience failure to respond to frontline therapy, and most fail to respond to the subsequent second-line TKI. Treatment guidelines for patients failing second-line therapy are lacking. This study aimed to determine the efficacy of TKIs as third-line therapy in a “real-world” clinical practice setting and identify factors favorably influencing the long-term outcomes of therapy.

**Methods:**

We have retrospectively analyzed the medical records of 100 patients with CP CML.

**Results:**

The median age of the patients was 51 (range, 21–88) years, and 36% of the patients were men. The median duration of the third-line TKI therapy was 22 (range, 1– 147) months. Overall, the rate of achieving complete cytogenetic response (CCyR) was 35%. Among the four patient groups with different levels of responses at baseline, the best results were achieved in the groups with any CyR at the baseline of third-line therapy. Thus, СCyR was reached in all 15 and 8/ 16 (50%) patients with partial cytogenetic response (PCyR) or minimal or minor CyR (mmCyR), respectively, whereas CCyR was detected only in 12/69 (17%) patients without any CyR at baseline (p < 0.001). Univariate regression analysis revealed that the factors negatively associated with CCyR achievement in thirdline TKI therapy were the absence of any CyR on first- or second-line TKI therapy (p < 0.001), absence of CHR prior to third-line TKI (p = 0.003), and absence of any CyR prior to third-line TKI (p < 0.001). During the median observation time from treatment initiation to the last visit [56 (4–180) months], 27% of cases progressed into accelerated phase or blast phase CML, and 32% of patients died.

**Discussion:**

Progression-free survival (PFS) and overall survival (OS) were significantly higher in patients with CCyR on third-line than in the group without CCyR on third-line therapy. At the last visit, third-line TKI therapy was ongoing in 18% of patients, with a median time of treatment exposure of 58 (range, 6–140) months; 83% of these patients had stable and durable CCyR, suggesting that patients without CHR at baseline and without CCyR at least by 12 months on third-line TKI should be candidates for allogeneic stem cell transplantation, third-generation TKIs, or experimental therapies.

## 1 Introduction

Tyrosine kinase inhibitors (TKIs) have significantly improved the outcomes of patients with chronic myeloid leukemia (CML), seemingly increasing their life expectancy to that of the general population (1). However, 35%–45% of patients fail to respond to first-line TKI within 10 years of therapy (2, 3). Most patients after first-line treatment discontinuation are switched to second-generation TKI. Overall, nilotinib, dasatinib, and bosutinib have demonstrated similar response rates, with probabilities of obtaining a complete cytogenetic response (CCyR) of approximately 50%. After 5 years of observation, up to 60% of patients discontinue second-line therapy mainly due to primary or secondary resistance (4–6). Treatment and response recommendations focus on first- and second-line therapy with first-generation (imatinib) or second-generation (nilotinib, dasatinib, or bosutinib) TKIs (7, 8). Currently, the ELN 2020 guidelines suggest the use of the third-generation TKI ponatinib as third-line therapy in patients without specific mutations rather than alternative second-generation TKIs. Patients with suboptimal response to two or more previous TKIs should be transferred to allogeneic stem cell transplantation (allo-SCT) (7). Allo-SCT may be considered for patients with *de-novo* blast phase (BP) CML, preferably after achieving some response to a TKI-based therapy, those with accelerated phase (AP) CML who are not responding well to current therapy, those who progressed to AP/BP CML while receiving TKI therapy, and those with resistance or intolerance to TKIs. It may also be used in patients with T315I mutation after an inadequate response to attempted ponatinib therapy (9). However, the possibilities for its implementation are limited due to the risks associated with age, comorbidities, and/or lack of matched donors. Globally, only a small proportion (≤1%) of patients with chronic phase (CP) CML undergo allo-SCT after failure of second-line therapy, while most patients continue to receive other TKIs (10).

It is very important to identify a group of patients in whom third-line therapy with TKIs is associated with equal or even better long-term outcomes than using allo-SCT. As a reference point for determining the effectiveness of third-line TKI, an assessment of the achievement of CCyR has been proposed. CyR, especially CCyR, has historically been and is currently associated with a significant survival advantage in patients with CP CML (11, 12).

This study aimed to evaluate the long-term efficacy of third-line therapy with available TKIs and identify the factors that could favorably influence CCyR achievement and survival prognosis.

## 2 Materials and methods

### 2.1 Patients

This multicenter retrospective observational study that aimed to evaluate the long-term efficacy of third-line therapy with TKI (third TKI) in patients with CP CML in real-life practice was conducted between 2019 and 2022. The inclusion criteria were as follows: 1) age ≥18 years, 2) CP CML (ELN criteria 2013), 3) treatment with available third-line TKI outside clinical trials with any reason of previous discontinuation, and 4) absence of CCyR at baseline. The exclusion criteria were 1) history of AP/BP and 2) prior allo-HSCT. All patients gave informed consent prior to participation. All study procedures were performed in accordance with the institutional and national ethical standards on human experimentation and with the Helsinki Declaration of 1975, as revised in 2008. Overall, 100 [men, *n* = 36 (36%)] patients from six centers of the Saint Petersburg and Leningrad region were included. The data of 73 patients were collected primarily in 2019 and updated in 2022. The data of the other 27 patients were collected in 2022. In all cases, the diagnosis of CML was confirmed using cytogenetic and molecular analysis (13). At the time of diagnosis and time of initiation of third-line TKI, the median age of the patients was 45 (range, 12–82) and 51 (range, 21–88) years, respectively. First-line TKI treatment for most patients was imatinib (*n* = 97, 97%). Nilotinib, dasatinib, imatinib, and bosutinib were used as second-line therapy in 69 (69%), 25 (25%), 1 (1%), and 5 (5%) patients, respectively. The median duration of CML from the time of diagnosis to first-line TKI and third-line TKI and from the initiation of first-line TKI and second-line TKI therapy to the initiation of third-line therapy was 2 (range, 1–245), 64 (range, 7–316), 48 (range, 7–156), and 17 (3–96) months, respectively. The main characteristics of the patients prior to third-line TKI initiation are shown in **Table 1**.

**Table 1:** Characteristics of patients on first- and second-line TKI therapy (*n* = 100).

Patient characteristics		First-line TKI	Second-line TKI	On first- and second-line TKI
Median duration (range)		22 (2–145)	14 (0.5–96)	46 (6–156)
Best responses, *n* (%)	No CHR	17 (17%)	13 (13%)	3 (3%)
	CHR without CyR	35 (35%)	36 (36%)	32 (32%)
	mmCyR	16 (16%)	17 (17%)	13 (13%)
	PCyR	9 (9%)	12 (12%)	17 (17%)
	CCyR	17 (17%)	14 (16%)	22 (22%)
	MMR	6 (6%)	8 (8%)	13 (13%)
	MR ≥4	0 (0%)	0 (0%)	0 (0%)
Time to best response, median (range), months		6 (1–67)	5 (1–24)	6 (1–61.5)
Reason for TKI withdrawal, *n* (%)	Resistance	90 (90%)	72 (72%)	72%
	Intolerance	10 (10%)	28 (28%)	24% continued therapy; 4% discontinued both TKIs due to resistance and intolerance
*BCR::ABL* mutations	Detected in evaluable patients any time on TKI, *n* (%)	10/33 (30%)	32/66 (48%)	40/91 (44%)
	Type of mutations	G250E—3 E255K—1 Е255V—2 М351Т—1 D363Y—1 H396P—1 Q252H+E255K—1	L248V—1 E355A—1 G250E—5 E255V—1 Y253F—1 Q252H—1 Y253H—6 F311C—2 T315I—6 F317L–4 F359C–1 T315I+Y253H—1 F317L+F359V—1 F317L+L248V—1	G250E—8 Е255V—3 М351Т—1 D363Y—1 H396P—1 F359C+Q252H+E255K—1 E255K+L248V—1 E355A—1 Y253F—1 Q252H—1 Y253H—6 F311C—2 T315I—6 F317L—4 T315I+Y253H—1 F317L+F359V—1 F317L+L248V—1

TKI, tyrosine kinase inhibitor; CHR, complete hematologic response; CyR, cytogenetic response; mmCyR, minimal or minor cytogenetic response; PCyR, partial cytogenetic response; CCyR, complete cytogenetic response; MMR, major molecular response; MR ≥4, deep molecular response ≥4 log.

Overall, any time before the initiation of third-line TKI therapy, *BCR::ABL* mutational analysis was performed in 91/100 (91%) patients. *BCR::ABL* mutations were evaluated on both first- and second-line TKIs only in 8/91 (8%) cases. Mutations were identified in 40/91 (43.9%) evaluable cases. After failure to respond to first-line TKI, 10 mutations were identified in 33 (30%) patients, with one patient having two mutations. Only five clinically significant mutations (G250E, E255K, Е255V, M351T, and Q252H) in seven patients were detected. After failure to respond to second-line therapy, TKI *BCR::ABL* kinase domain point mutations were identified in 32/66 patients (48%), with mutations being repeatedly identified in two patients following first-line therapy. Only eight clinically significant mutations in 27 patients (G250E, Е255V, Q252H, T315I, Y253H, F317L, F359C, and L248V) were detected. The T315I mutation was detected in 7/91 (8%) patients.

Additional chromosomal aberrations were detected in 19/100 (19%) patients at any time prior to third-line therapy.

At the baseline of third-line TKI therapy, 35 (35%), 34 (34%), 16 (16%), and 15 (15%) patients did not have a complete hematologic response (CHR) and had CHR without cytogenetic response (CyR), minor or minimal CyR (mmCyR), and partial CyR (PCyR), respectively. No patients with CCyR or deeper responses at baseline were included in the study according to the inclusion criteria.

### 2.2 Statistical analysis

The cumulative rate of CCyR achievement on third-line TKI was assessed as the primary efficacy endpoint of third-line TKI. All decimal numbers were converted to the nearest whole number. CCyR was defined as 0% of Ph+ metaphases, with at least 20 metaphases required, according to bone marrow cytogenetics or 2-log reduction of molecular response (MR2), with a *BCR::ABL/ABL* ratio of ≤1% in peripheral blood by reverse transcription quantitative real-time polymerase chain reaction test (14).

To test the type of distribution of quantitative variables, the Shapiro–Wilk *W*-test was used. Variables did not have a normal distribution in any of the cases. Continuous variables were reported as medians and ranges. Categorical variables were reported as absolute values and percentages. Differences between categorical variables were calculated using the *χ* ^2^ test, and differences between continuous variables were calculated using the Mann–Whitney *U* test.

Overall survival (OS) was defined as the time from the initiation of third-line TKI therapy to death. Progression-free survival (PFS) was defined as the time from the initiation of third-line TKI therapy to disease progression or death, whichever occurred first. Event-free survival (EFS) was defined as the time from the initiation of third-line therapy to therapy withdrawal, disease progression, or death, whichever occured first (15). OS and PFS were calculated using the Kaplan–Meier method. The log-rank test was used to assess the significance of differences in survival between the patient groups.

The influence of possible risk factors on CCyR achievement was assessed by univariate analysis.

## 3 Results

At the time of the last data collection, the median duration of third-line TKI therapy was 22 (range, 1–147) months, and the median time of follow-up from the initiation of third-line TKI and from diagnosis to the last visit was 56 (range, 4–180) months and 125 (range, 27–434) months, respectively. Only four patients were lost to follow-up after a median observation time of 29 (range, 5–79) months.

Patients received dasatinib, nilotinib, bosutinib, or ponatinib as third-line therapy in 58 (58%), 22 (22%), 17 (17%), and 3 (3%) cases, respectively.

### 3.1 Response to therapy

#### 3.1.1 CHR

At baseline, 65 patients had CHR, and 26/35 (74%) patients who did not have CHR at baseline achieved it by 3 months of therapy. CHR was subsequently lost in 9/26 (35%) and 5/65 (8%) patients without and with CHR at baseline, respectively (*p* = 0.002).

#### 3.1.2 CCyR

With a median time on third-line TKI therapy of 22 (range, 1–147) months, CCyR (or MR2) was achieved in 35/100 (35%) patients. The median time to response was 6 (range, 2–22) months. Most patients (23/35; 66%) obtained CCyR within 6 months of treatment. No patients with prolonged treatment of third-line TKI reached CCyR after 24 months.

The influence of the baseline level of response to CCyR in patients undergoing third-line TKI therapy was assessed. The median time on third-line TKI therapy for patients without CHR, with CHR but no CyR, and with any CyR (mmCyR or PCyR) was 20 (range, 1–95), 18 (range, 1–147), 34 (range, 5–140), and 32.5 (range, 3.5–121) months, respectively. CCyR was reached in 30/65 (46%) and 5/35 (14%) patients with and without CHR at baseline (*p* = 0.02). No differences in CCyR achievement were observed in patients without CHR and with CHR but no CyR (*p* > 0.05). Meanwhile, CCyR was significantly higher in patients with any baseline CyR than in patients with no CyR with or without CHR (*p* < 0.05). Moreover, all patients with any CyR at baseline reached CCyR during therapy (*p* = 0.013). The rate of CCyR in the patient groups is presented in **Table 2**.

**Table 2:** The cumulative rate of CCyR in patients undergoing third-line TKI therapy.

Cumulative rate of CCyR, *n* (%)	All patients (*n* = 100)	Baseline response			
		No CHR *n* = 35	CHR but no any CyR *n* = 34	mmCyR *n* = 16	PCyR *n* = 15
Any time	35 (35%)	5 (14%)	7 (20.5%)	8 (50%)	15 (100%)
By 6 months	23 (23%)	3 (9%)	5 (15%)	5 (31%)	10 (67%)
By 12 months	30 (30%)	3 (9%)	7 (21%)	6 (37.5%)	14 (93%)

**Figure 1:**
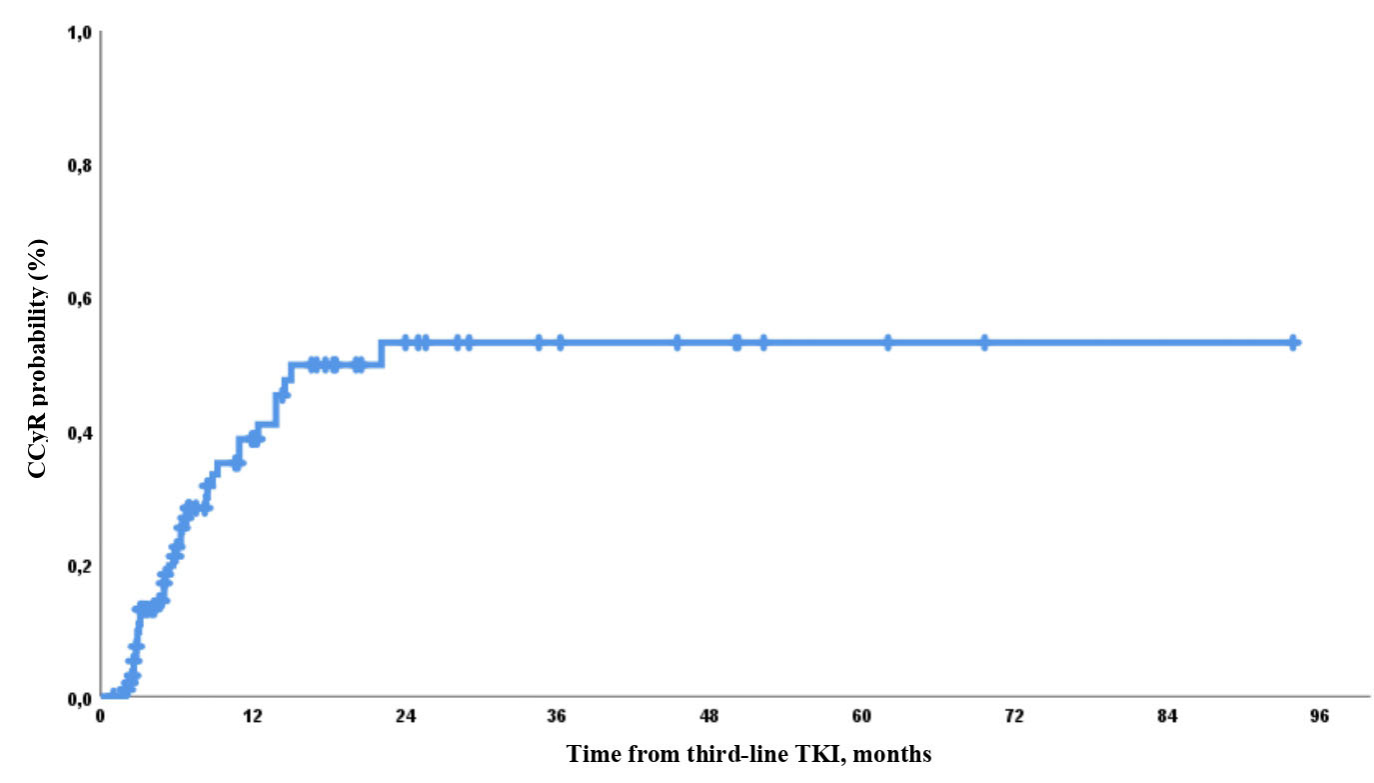
Probability of complete cytogenetic response (CCyR) achievement during third-line tyrosine kinase inhibitor (TKI) therapy.

CCyR was also higher in patients without clinically significant *BCR::ABL* mutations than in those with such mutations. Thus, CCyR was obtained in 8/34 (24%) and 27/57 (47%) evaluable patients with and without clinically significant mutations of *BCR::ABL*, respectively (*p* = 0.024). The probability of CCyR achievement by 12 and 24 months was 41% and 54%, respectively (**Figure 1**).

**Figure 2:**
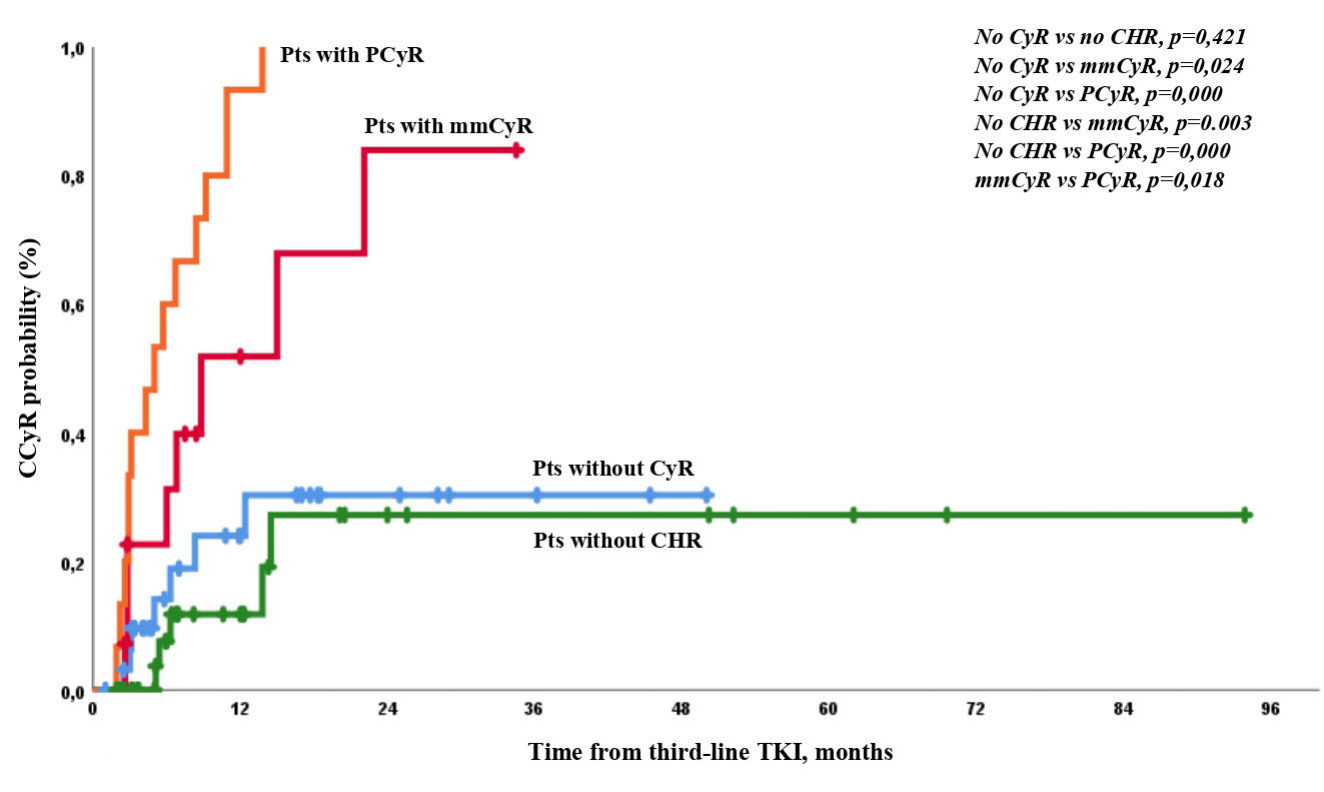
Probability of CCyR achievement in the different groups of patients according to the level of response at baseline.

The expected CCyR by 12 months according to initial response was 12%, 31%, 52%, and 94% in patients without CHR, with CHR but no CyR, with mmCyR, and with PCyR, respectively (**Figure 2**).

Loss of CCyR was observed in 13/35 (37%) patients after a median time of 22 (range, 2–46) months. By the last visit, CCyR was sustained in 2/5 (40%), 4/7 (57%), 5/8 (62,5%), and 11/15 (73%) patients with no CHR, with CHR but no CyR, and with any CyR (mmCyR or PCyR), respectively. No statistical differences were observed among patients with different baseline levels of response. In patients with sustained CCyR, the median duration of response was much longer and reached 49 months (range, 2–144.2; *p* < 0.05). Time to CCyR achievement was shorter in patients with stable response than with loss of CCyR [4 (range, 2–22) *vs*. 8 (range, 2.5–15) months, respectively; *p* > 0.05], though this difference was not statistically significant. The rate of CCyR loss was nearly similar in patients that achieved CCyR by 6 months of therapy (6/23; 26%) and those that achieved it later (7/12, 58%; *p* = 0.06).

Among patients with CCyR, 17/35 (49%) obtained a major molecular response (MMR) or better molecular responses. At the last visit, all patients except three had stable MMR or better molecular response. CCyR was lost only in 1/17 (6%) and 12/18 (67%) patients with and without MMR, respectively (*p* = 0.001).

### 3.2 Factors influencing the probability of CCyR achievement

Results of the univariate regression analysis (**Table 3**) revealed that the factors negatively associated with CCyR achievement during third-line TKI were as follows:

- Absence of any CyR during first- or second-line TKI therapy (*p* < 0.001)
- Absence of CHR prior to third-line TKI therapy (*p* = 0.003)
- Absence of any CyR prior to third-line TKI therapy (*p* < 0.001)

**Table 3:** Univariate regression analysis for the risk of not achieving a CCyR on third-line TKI therapy.

Prognostic factor	OR	95% CI	*p*-value
Sex, male	1.36	0.58-3.34	0.485
Age at start of third-line TKI (per 10 years)	1.1	0.83-1.48	0.514
Age at start of third-line TKI >60 years	1.19	0.48-3.12	0.709
Time from diagnosis to third-line TKI (per year)	0.997	0.99-1.004	0.395
Time from first- to third-line TKI (per year)	0.999	0.987-1.011	0.873
Absence of CyR on first-line or second-line TKI	0.063	0.01-0.23	<0.001*
Absence of CHR prior to third-line TKI	0.194	0.06-0.53	0.003*
Absence of CyR prior to third-line TKI	0.073	0.025-0.19	<0.001*
Mutations on second-line TKI	1.17	0.38-3.63	0.777

OR, odds ratio.

*Indicates statistical significance (p < 0.05).

### 3.3 Progression to advanced phases and PFS

During the median observation time of 56 (range, 3.5–179.5) months from treatment initiation to the last visit, 27/100 (27%) patients progressed into AP/BP CML. During third-line TKI treatment, AP/BP was registered in 10/27 (37%) patients, with a median time to progression of 12 (range, 4–39) months. In 17/27 (63%) patients with AP/BP after third-line TKI discontinuation, the median time to progression was 28 (range, 9–110) months. More than a quarter (7/27; 26%) of the progressions occurred within the first year of third-line TKI therapy. As for other cases, 4/27 (15%), 7/27 (26%), 5/27 (19%), and 4/27 (15%) were diagnosed with AP/BP CML during the second year, third year, between 3 and 5 years, and beyond 5 years of observation, respectively.

The median observation time of patients without CHR, with CHR but no CyR, and with any CyR (mmCyR or PCyR) was 44 (range, 5–169), 58 (range, 8–180), 63 (range, 5–140), and 56 (range, 4–121) months, respectively. As expected, most cases of AP/BP CML developed in patients without CHR at baseline (*n* = 17/35; 48.5%). A comparable proportion of patients without CHR at baseline progressed regardless of CHR achievement on third-line TKI therapy [achievement, 5/9 (56%); non-achievement, 12/26 (46%); *p* = 0.6]. The transformation rate was lower in the CHR but no CyR group (8/34; 23.5%) than in the no CHR group [17/35 (48.5%); *p* = 0.031]. Patients with any CyR at baseline had similar rates of progressions. Thus, AP and BP were diagnosed in 2/16 (12.5%) and 0/15 (0%) patients with any CyR, respectively (*p* = 0.191).

**Figure 3:**
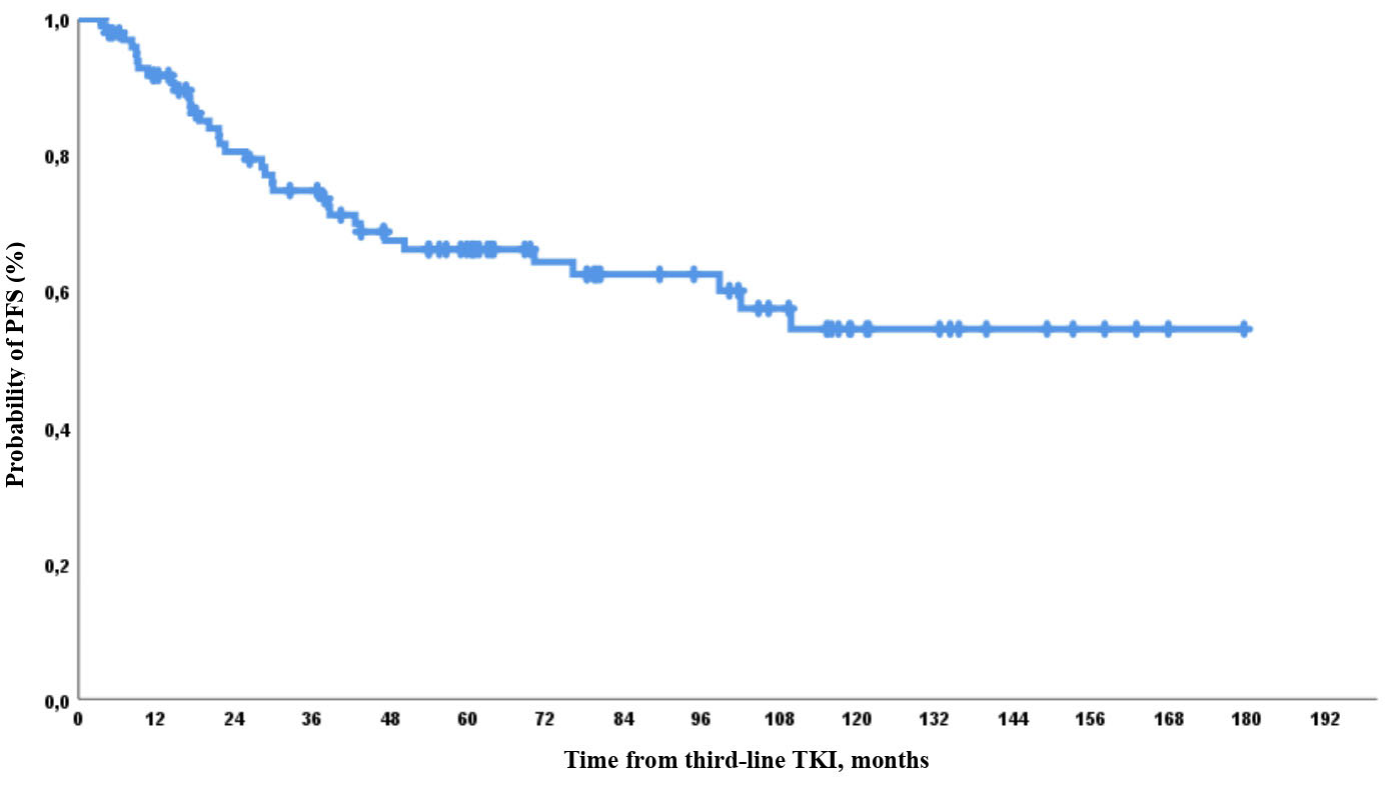
Progression-free survival (PFS) on third-line TKI therapy.

Thirty-five events were included in the analysis of PFS: 27/100 (27%) patients progressed, and 8 deaths occurred owing to the following reasons: transplant-related causes (*n* = 3) and other reasons (*n* = 5), including cardiovascular disease (*n* = 3), other secondary neoplastic processes (*n* = 1), and SARS-CoV-2 infection (1). The 1-, 2-, 3-, and 5-year PFS rates were 92%, 83%, 77%, and 68%, respectively (**Figure 3**).

**Figure 4:**
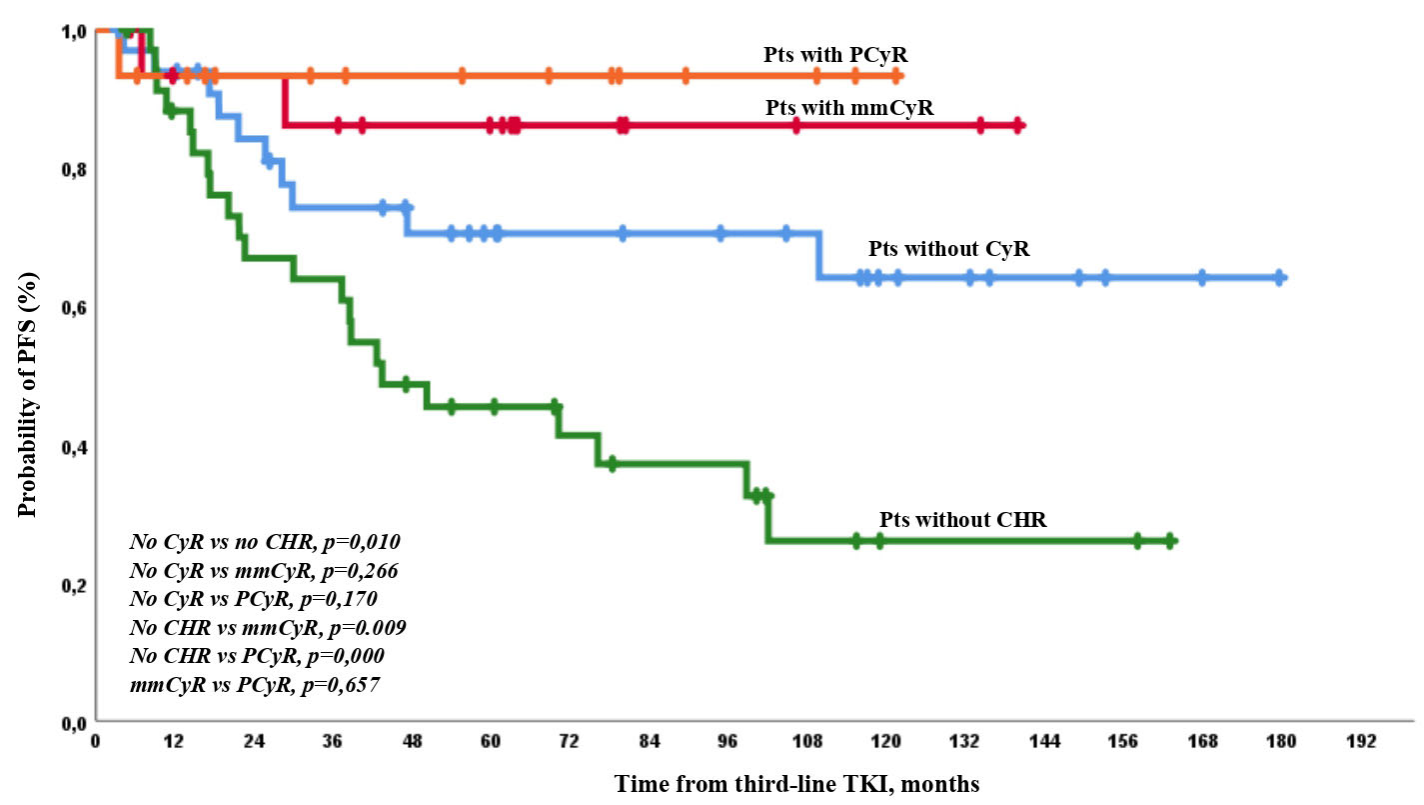
PFS on third-line TKI therapy according to the depth of response at baseline. There were only 1/35 (3%) and 26/65 (40%) cases of AP/BP among patients who achieved or did not achieve CCyR on third-line TKI therapy (*p* = 0.0001).

The expected PFS was similar and was very high among patients with any CyR at baseline. It was lower in patients with CHR but no CyR than in other patients. There were no significant differences between patients with different levels of cytogenetic resistance at baseline. The worst PFS was observed in patients with hematologic resistance (**Figure 4**).

**Figure 5:**
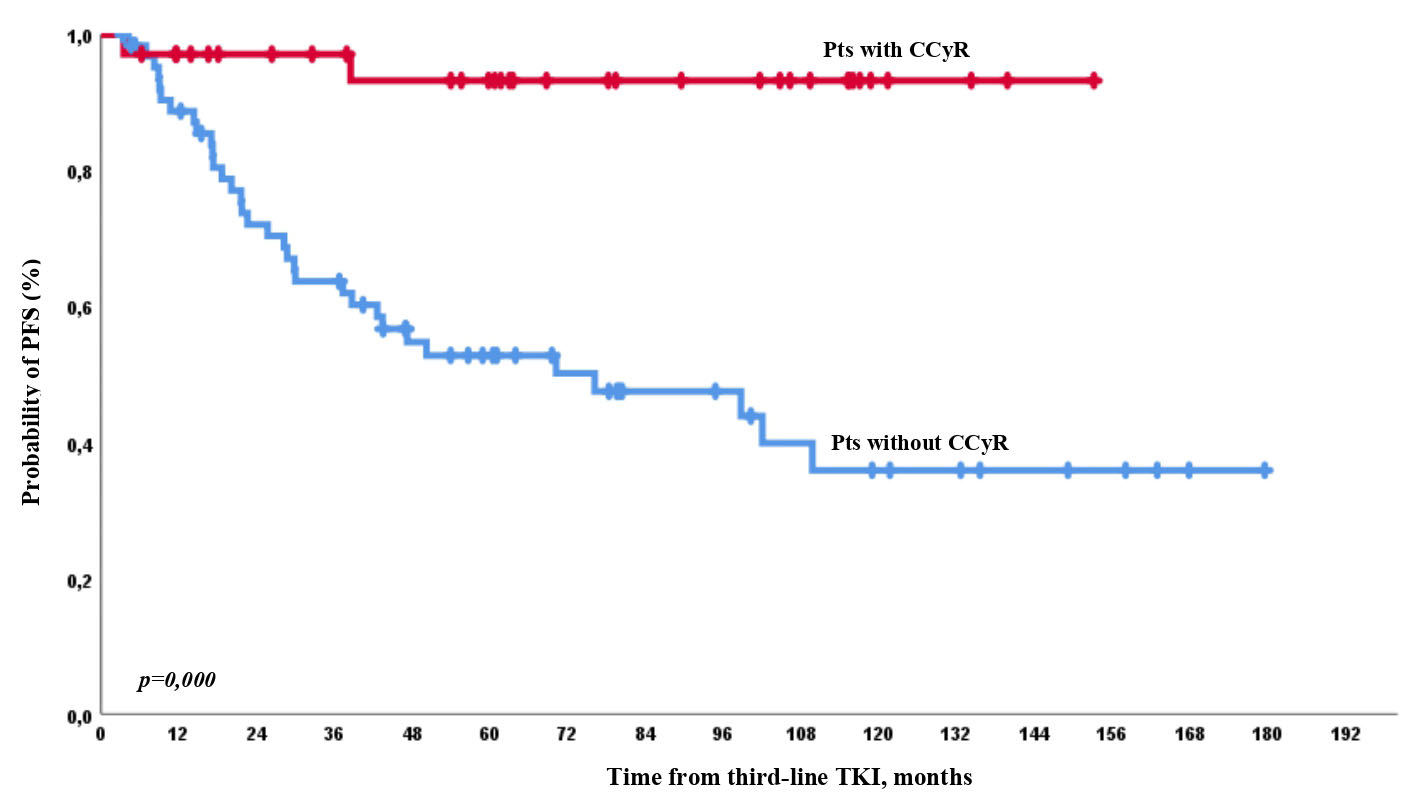
PFS on third-line therapy according to CCyR achievement on third-line TKI.

PFS events occurred in 2 and 33 patients with or without CCyR on third-line TKI, respectively. The 1-, 2-, 3-, and 5-year PFS rates were 97%, 97%, 97%, and 93% in patients with CCyR achievement on treatment and 88%, 72%, 63%, and 52% in patients with no CCyR on treatment, respectively, with significant differences at any time point (**Figure 5**).

### 3.4 OS

With the median observation time from the beginning of third-line therapy to the last visit of 56 (range, 3.5–179.5) months, 32/100 (32%) deaths occurred, 21 of which were due to CML progression, 6 occurred after complications of allo-SCT (*n* = 3, allo-SCT was performed due to BP), and 5 occurred for other reasons [cardiovascular disease, *n* = 3; other secondary neoplastic processes, *n* = 1; and SARS-CoV-2 infection, *n* = 1 (1)]. Drug-related toxicity did not cause any deaths (**Table 4**). The causes of death in different groups are shown in **Table 4**.

**Table 4:** Causes of death according to the depth of response at baseline.

Cause of death	Patients without CHR	Patients without CyR	Patients with mmCyR	Patients with PCyR	All	*р*
Progression	13	8		0	21 (65%)	<0.01
Complications after allo-SCT	5	0	1		6 (19%)	
Deaths from other causes	2	2	0	1	5 (16%)	
All	20 (63%)	10 (31%)	1 (3%)	1 (3%)	32 (100%)	

**Figure 6:**
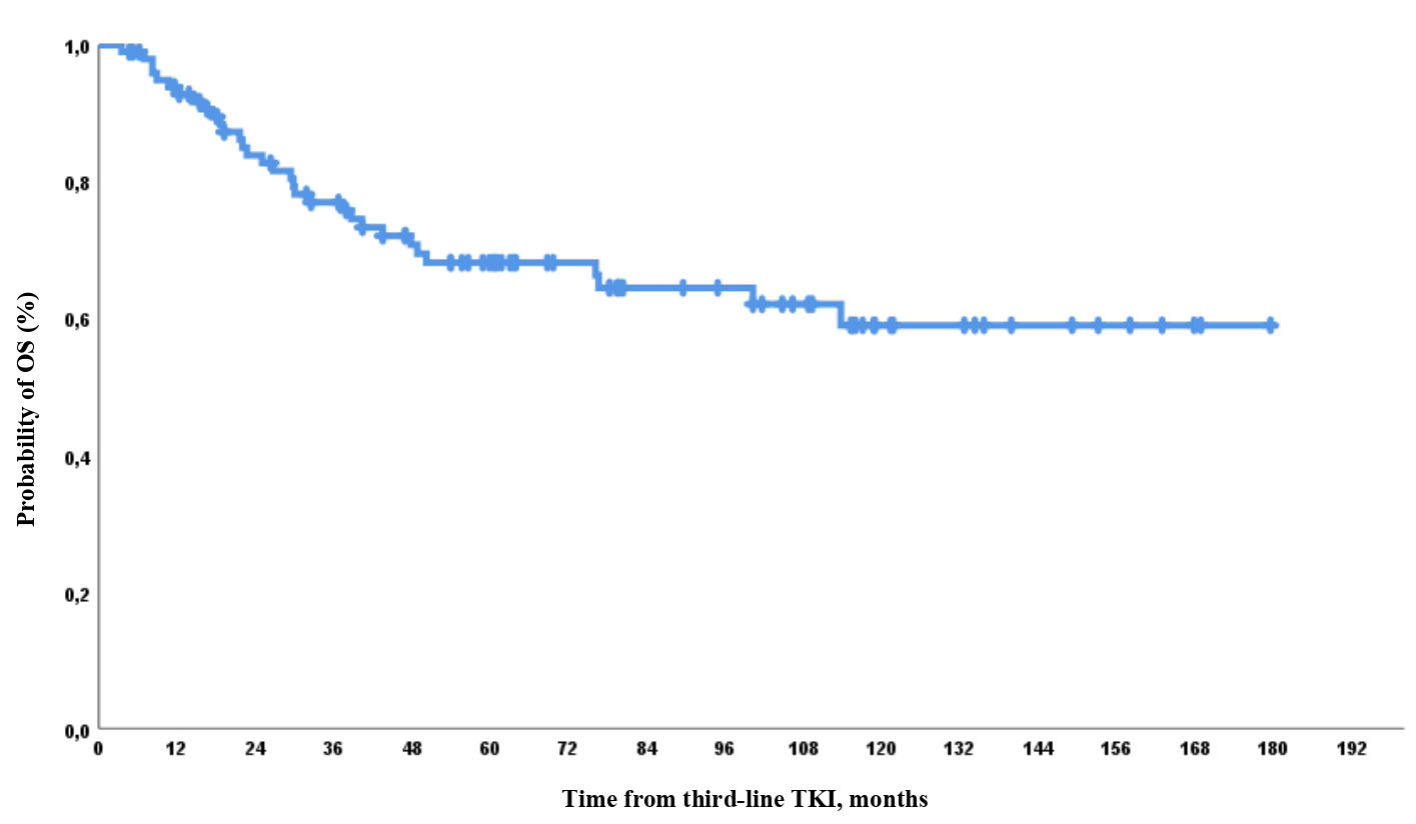
Overall survival (OS) on third-line therapy.

Deaths while on third-line TKI were observed in 5/32 (16%) cases, with a median time to death of 30 (range, 4–50) months. After third-line therapy discontinuation, there were 27/32 (63%) cases of death, with a median time to mortality of 25 (range, 7–114) months. Deaths were observed in 6/100 (6%), 14/100 (14%), 20/100 (20%), and 28 (28%) cases by 12, 24, 36, and 60 months from third-line TKI, respectively. OS was 92%, 83%, 77%, and 68% by 12, 24, 36, and 60 months of observation, respectively (**Figure 6**).

After a nearly equal observation time from third-line treatment, it was found that the rate of death was lower in the group with any depth of CyR than in the group with no CyR but with CHR and the group without CHR at baseline [2/31 (6.5%) *vs*. 10/34 (29%) *vs*. 20/35 (57%), *p* < 0.01]. Simultaneously, patients with mmCyR and PCyR have a similar mortality rate of 1/16 (6%) *vs*. 1/15 (7%) (*p* = 0.9), respectively.

**Figure 7:**
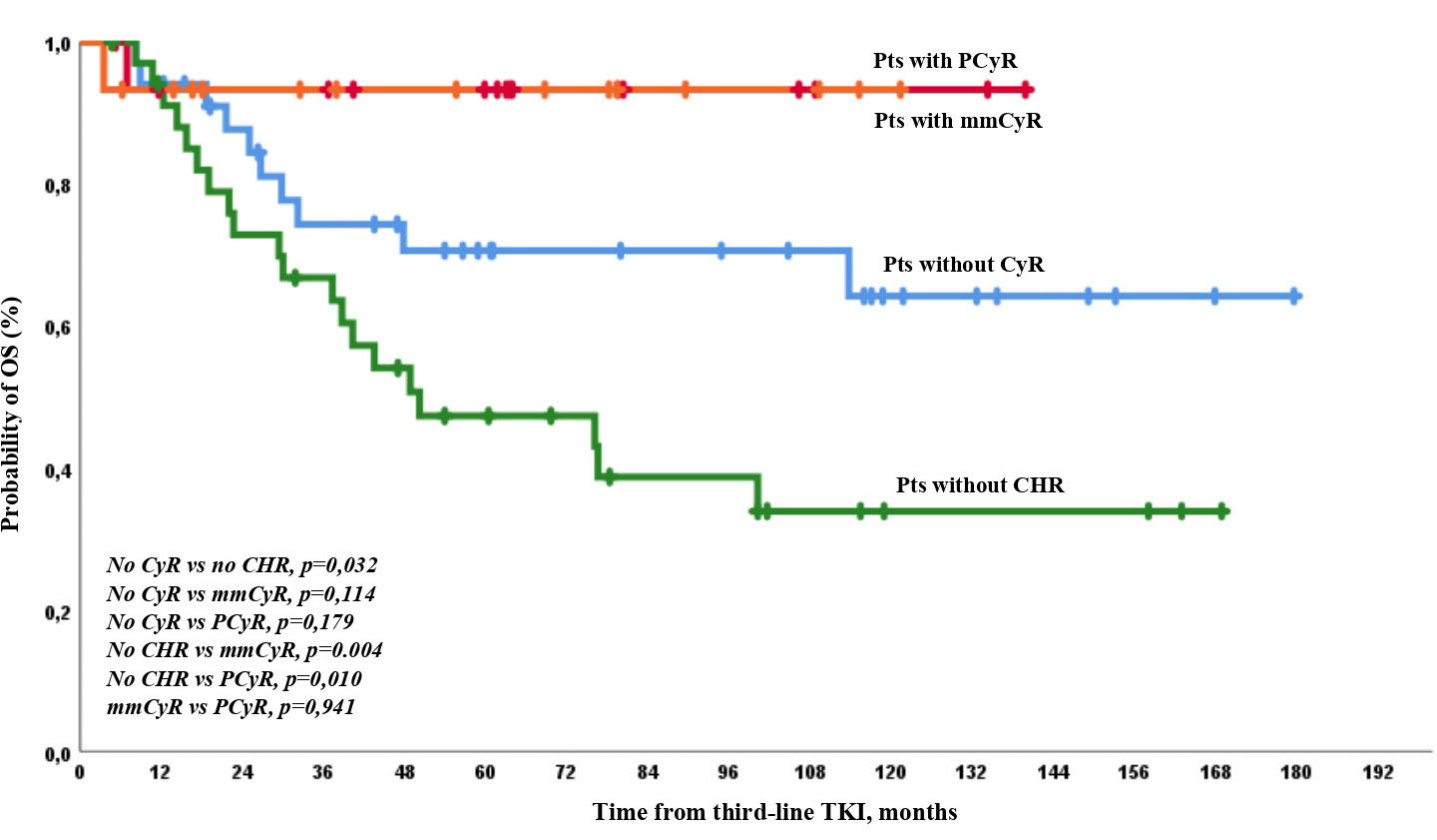
OS on third-line therapy according to the depth of response at baseline.

The expected OS was the lowest in patients with no initial CHR. It was higher in patients with any CyR than in patients with CHR but no CyR (**Figure 7**).

**Figure 8:**
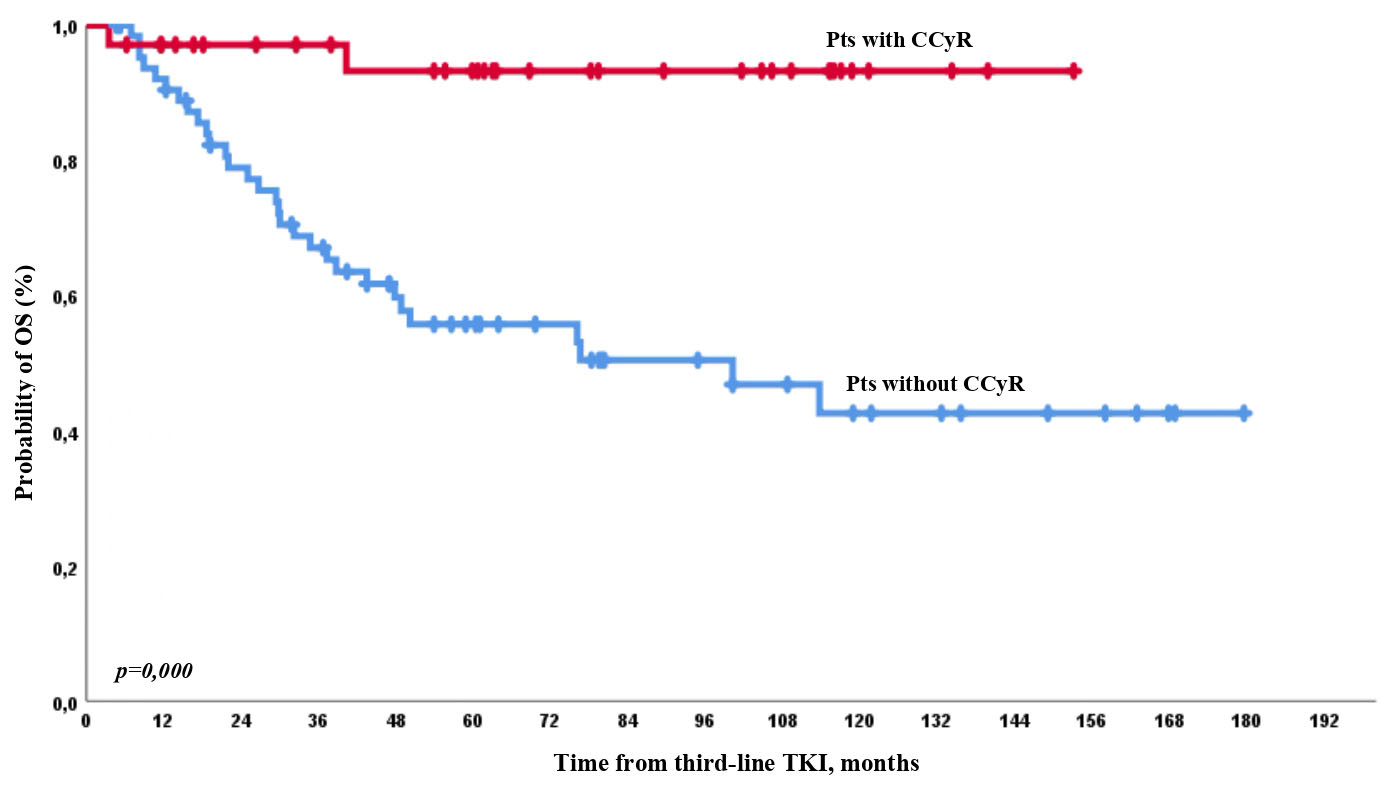
OS on third-line therapy according to CCyR achievement on third-line TKI.

No differences in mortality rate between patients without CHR at baseline but who reached and did not reach CHR during third-line therapy were observed [14/26 (54%) and 6/9 (67%), respectively, *p* = 0.5]. Meanwhile, there were only 2/35 (6%) and 30/65 (46%) deaths among patients who achieved and did not achieve CCyR at any time on third-line TKI (*p* = 0.0001). OS was 97% *vs*. 90%, 90% *vs*. 77%, 90% *vs*. 67%, and 93% *vs*. 55% by 12, 24, 36, and 60 months of observation, respectively, between patients with CCyR who achieved or did not achieve CCyR during therapy (**Figure 8**).

### 3.5 EFS and patients’ current status

With a median time of third-line TKI of 22 (range, 1.0–147) months, third-line TKI was discontinued in 82/100 (82%) patients in the whole group. TKI withdrawal due to resistance, death unrelated to CML, TKI toxicity, and treatment-free remission occurred in 66/82 (80%), 5/82 (6%), 10/82 (12%), and 1/82 (1%) cases, respectively.

EFS was defined as the time from the initiation of third-line therapy to therapy withdrawal or to disease progression or death, whichever occured first, as described earlier. Therapy discontinuation during deep molecular response for treatment-free remission was censored at the time of drug withdrawal. There were 67/100 (67%) withdrawals, 10/100 (10%) progressions, and 5/100 (5%) deaths. Thus, 18/100 (18%) patients continued third-line TKI therapy.

**Figure 9:**
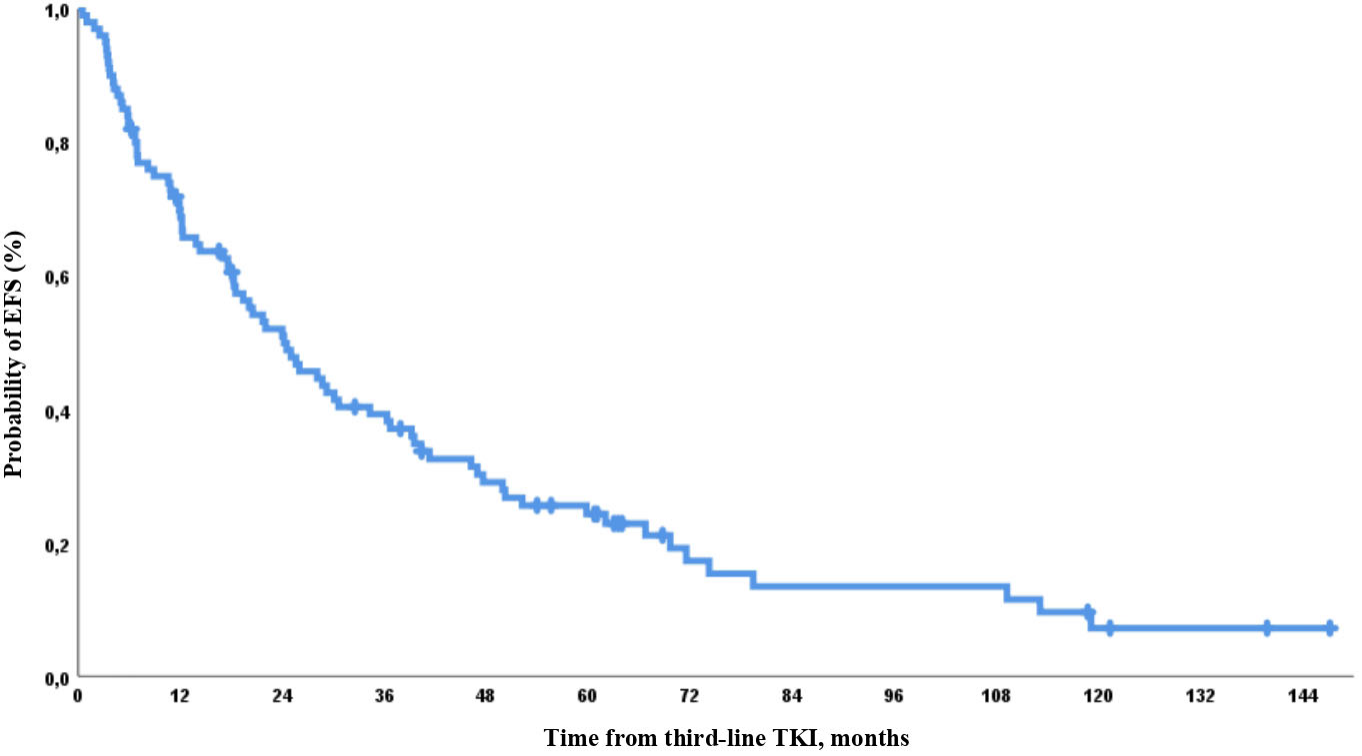
EFS on third-line therapy in the whole group.

EFS by 12, 24, 36, and 60 months was 65%, 48%, 37%, and 24%, respectively (**Figure 9**).

**Figure 10:**
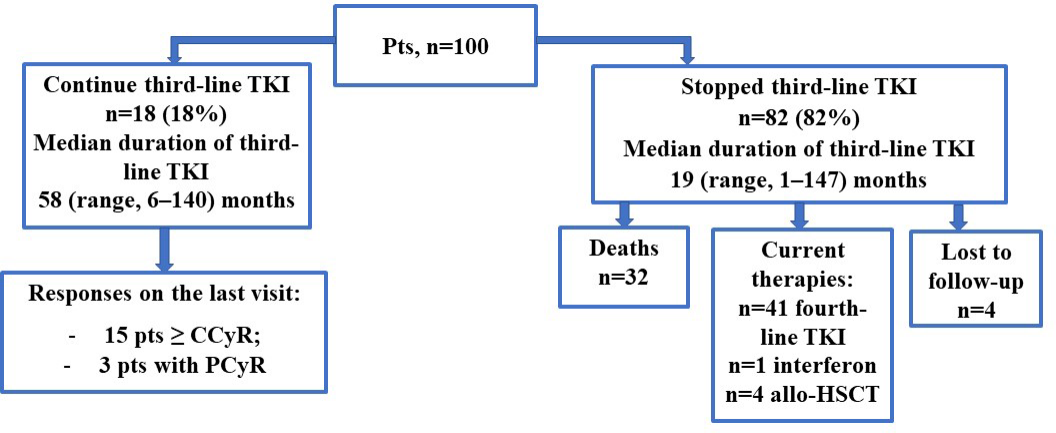
Long-term outcomes in the whole group on and after third-line TKI.

There were 35/100 (35%) with CCyR achievement on third-line TKI, 20/35 (57%) of whom discontinued third-line therapy. The reasons for discontinuation were as follows: progression in 1/20 (5%), death because of other reasons (cardiovascular disease complications) in 1/20 (5%), treatment-free remission in 1/20 (5%), secondary molecular resistance in 1/20 (5%), secondary cytogenetic resistance in 13/20 (65%), and toxicity in 3/20 (15%). At the last visit among patients with CCyR, 9/15 (60%) patients had an MMR or better molecular response. The long-term outcomes are presented in **Figure 10**.

## 4 Discussion

Most patients were treated with dasatinib and nilotinib as third-line TKI. Bosutinib was the treatment of choice only in 17% of patients. The drug was registered in our country much later than other TKIs and was available for fewer patients. Ponatinib was not registered in the country at the time of data collection. Only three patients were provided with ponatinib *via* different programs outside of clinical trials. Owing to recent advances in TKI therapy, patients with CP CML have a low incidence of progression to AP/BP and CML-related death. Moreover, up to 30% of patients with a stable deep molecular response have a chance of treatment-free remission. Meanwhile, up to 45% and 60% of patients, respectively, discontinue first- and second-line TKI therapy due to resistance or intolerance. Current guidelines consider ponatinib and allo-SCT as a third-line strategy, especially for patients with TKI resistance, but in real-life practice, nearly all patients are switched to third-line TKI therapy with second-generation TKI because of different socioeconomic status and inaccessibility of third-generation TKI. In our previous study (16), only 21% of new cases of CCyR among patients with CP CML undergoing second-generation TKI therapy continued to third-line therapy, and 21/53 (40%) patients experienced drug withdrawal in less than 2 years of observation. The 2-year OS was 67%. All patients with major CyR on third-line TKI were in stable CP and alive during observation (16). In our current retrospective study, CCyR was a significant marker for both PFR and OS. Among the 35 patients with CCyR, only one patient died due to progression to AP/BP and another one died for a reason not related to CML. Both PFS and OS were significantly higher at any time point in patients with CCyR than in patients with no CCyR during third-line treatment. In these groups of patients, the 5-year PFS and OS were 93% *vs*. 52% and 93% *vs*. 55%, respectively (*p* < 0.05). According to the results of our study, 27/100 (27%) patients progressed to advanced phases. Progression to BP occurred mostly among patients without CHR at baseline [*n* = 17/35 (49%)]. There were only two progressions in the mmCyR group [2/16 (12.5%)]. Disease progression did not occur among patients with PCyR at baseline. Similar results were shown by Garg et al. (17), where transformation to AP/BP was reported in 12/48 (25%) patients. Among patients with AP/BP progression, most [8/12 (67%)] did not achieve any CyR prior to third-line TKI therapy (17). In an article by Ribeiro et al. (18), among 18 patients with CP CML, only 3/18 (16%) progressed to BP on third-line therapy. Khan et al. (19) reported that the depth of response to therapy (both CCyR and MMR) was associated with improved OS in the univariate analysis. In a multivariate analysis, MMR was a favorable prognostic marker for OS (19). In our study group, MMR or better molecular responses were reached in 17/35 (49%) patients with CCyR. All patients were alive at the last visit, and 14/17 (82%) patients had stable MMR or better molecular response. CCyR loss was observed only in one out of 17 (6%) cases among patients with MMR achievement, as compared with 12/18 (67%) cases of CCyR without MMR achievement (*p* = 0.001). As only two patients died in the CCyR group, no difference was considered present between patients with and without MMR achievement during observation. In our observation, 35/100 (35%) patients reached CCyR. In our study, loss of CCyR on third-line therapy occurred in 13/35 (37%) patients. Ongoren et al. (20) reported that most patients on third-line TKI therapy achieved CCyR [11/21 (52%)], and only one patient lost CCyR on third-line therapy [1/11 (9%)] (20).

While CCyR was a strong surrogate marker of survival on third-line TKI, we have attempted to search for factors that influenced CCyR achievement. The baseline level of response and also prior responses and mutations were studied as prognostic factors for CCyR achievement. Among the four patient groups with different responses at baseline, the best results were achieved in the groups with any CyR at baseline. Thus, CCyR was reached in 23/31 (74%) patients with any level of CyR compared with 12/69 (17%) patients without any CyR at baseline (*p* = 0.0001). These results were compatible with the outcomes of the study of Ibrahim et al. (21), which showed that the rate of the 30-month cumulative incidence of CCyR in subgroups with and without any CyR was 71% and 0%, respectively (*p* = 0.0005). The significance of the depth of cytogenetic response before third-line therapy was also assessed in the study of Bosi et al. (22), where among the 13 patients who failed to respond to first- and second-line therapy, six with cytogenetic response before third-line therapy achieved a deeper cytogenetic response and higher overall survival on third-line TKI therapy.

We performed univariate analyses to identify pretreatment factors that predicted complete cytogenetic response. In the univariate analysis, the factors found to have a significant influence on the probability of achieving a complete cytogenetic response were age at third-line therapy, CyR on first- or second-line therapy, CyR before third-line therapy, and CHR before third-line therapy. The presence of kinase domain mutations prior to third-line therapy did not affect the probability of achieving a complete cytogenetic response, as discussed previously. For the multiple regression analysis, we included also the time from the diagnosis to third-line therapy as a factor that reliably affects the achievement of a cytogenetic response according to the data of global clinical practice (23).

Of note, most patients with CCyR on third-line TKI reached it within the first 12 months of therapy. Thus, the cumulative incidence of CCyR was 66% and 86% by 6 and 12 months of treatment, respectively.

Prior cytogenetic response is the most robust positive prognostic factor identified in patients with CML receiving TKIs in third-line therapy. This fact has been confirmed by our research, in addition to other studies. Ibrahim et al. (21) reported that CCyR on first- or second-line therapy was the only factor that affected the outcomes of third-line therapy (21). Third- and fourth-line therapy with bosutinib was also successful in the subgroup with any cytogenetic response at baseline. CCyR was achieved in 94% (31/33) of patients with any CyR at baseline in contrast, and the probability to achieve CCyR in the group without any CyR at baseline was 25% (7/28) (24). Again, in the study of Russo Rossi et al. (25), patients who did achieve a CyR on imatinib or had low and intermediate Sokal risk had a higher probability of achieving CyR with third-line TKI therapy (*p* < 0.001).

Baseline factors also influenced PFS and OS. The 5-year OS on third-line therapy in our study was 68%. Ribeiro et al. (18) reported higher 5-year OS rates among patients with CP CML (86%) than among patients with AP/BP CML. In the study of Cortes et al. (26), the 4-year OS among patients treated with third-line bosutinib was 78%. We could speculate that some patients took a longer time to reach third-line TKI, had more clinically significant *BCR::ABL* gene mutations, especially with T315I, or had other prior unfavorable factors compared with the patients in the aforementioned study, although there were not enough data for comparison. When comparing baseline factors, hematologic resistance was associated with the worst outcomes pertaining to PFS and OS, although we did not find any statistical differences between groups with any level of cytogenetic responses at third-line TKI initiation. Most of our patients who failed to respond to therapy were switched to third-generation TKIs as their fourth or even fifth form of therapy within clinical trials; accordingly, we suspect that the high survival rate may be associated with subsequent therapies. Although we did not include these data in this analysis, many patients continued conservative TKI therapy without any CyR but were still in CP. We can speculate that patients with unfavorable biological changes, including genetic aberrations outside of *BCR::ABL*, progressed earlier while on first- or second-line TKIs and did not reach third-line treatment. Accordingly, a population-based study to evaluate the real unmet needs of patients on third-line therapy is warranted. Another obvious limitation of our study is its retrospective nature; accordingly, there were some gaps in data for some indicators and analysis. Moreover, the sample size was small; therefore, some of the analyses lacked statistical power to draw a clear conclusion. However, in our opinion, we have obtained interesting findings through the current retrospective research, which may allow us, along with the data of other authors, to define a therapeutic strategy in patients who responded to the first two generations of TKI therapy. In our observation, long-term treatment outcomes are not favorable as more than 80% of the patients discontinued third-line TKIs mainly due to resistance. These results can be improved by using ponatinib or asciminib.

## 5 Conclusion

TKI has revolutionized CML management, and the availability of different TKIs provides patients with some alternatives after failure to respond to first- and second-line therapy (27). The data reviewed here suggest that independent prognostic factors may be helpful for the identification of patients who are unlikely to achieve CCyR response on TKIs after failure to respond to imatinib or a prior second-generation TKI. The cytogenetic response prior to third-line therapy, as well as some degree of cytogenetic response during first- or second-line therapy, was proven to be highly informative. Patients with these two criteria had a much higher probability of achieving CCyR on third-line therapy. Patients with CCyR and those without baseline hematologic resistance had better PFS and OS than the remaining patients. Patients with any level of CyR response on previous TKIs, especially with PCyR, are seemingly good candidates for continued conservative therapy, even with second-generation TKIs. These patients should be closely monitored for CyR and molecular responses. Patients without CCyR by 6 months or especially 12 months on third-line TKI, as well as patients with unfavorable baseline prognostic factors, especially those without CHR, should be switched to other available therapies, including allo-SCT, third-generation TKIs, or experimental drugs.

## Data availability statement

The datasets presented in this article are not readily available because all patient data is anonymized. Requests to access the datasets should be directed to chitanava_tv@almazovcentre.ru.

## Author contributions

EL, VS, EMo, SV, and YV contributed to the conception and design of the study. TC organized the database. TC and IuM performed the statistical analysis. TC, IuM, VS, SV, IM, YV, EE, EM, AP, TM, RV, EK, NI, NM, ND, TS, NS, OK, ES, NL, JA, DM, EM, and EL provided research materials. TC wrote the first draft of the manuscript. EL, IuM, VS, SV, and EM wrote sections of the manuscript. All authors contributed to the article and approved the submitted version.
